# Delivery of silver sulfadiazine and adipose derived stem cells using fibrin hydrogel improves infected burn wound regeneration

**DOI:** 10.1371/journal.pone.0217965

**Published:** 2019-06-13

**Authors:** Jaideep Banerjee, Shanmuganathan Seetharaman, Nicole L. Wrice, Robert J. Christy, Shanmugasundaram Natesan

**Affiliations:** Combat Trauma and Burn Injury Research, US Army Institute of Surgical Research, Ft. Sam Houston, TX, United States of America; University of Massachusetts Medical School, UNITED STATES

## Abstract

Infection control is necessary for improved burn wound regeneration. In this study contact burn wounds were induced on the dorsum of the rats and were infected with *Pseudomonas aeruginosa* (10^7^cfu/ml of saline) and left overnight (12–14 hours) to establish the infection. After 12 hours, the wounds were treated with PEGylated fibrin hydrogel containing 50 mgs of silver sulfadiazine (SSD) loaded chitosan microsphere (SSD-CSM-FPEG). On day 9, SSD-CSM-FPEG treated burn wounds further received adipose derived stem cell (5×10^4^ ASCs cells/ml) embedded in PEGylated fibrin hydrogel. Wounds were assessed for the healing outcomes such as neovascularization, granulation tissue formation, wound closure and collagen maturation. Analysis of bacterial load in the burn wound biopsies, demonstrated that SSD–CSM-FPEG significantly reduced bacterial infection, while overt infection was still observed in the untreated groups on day 14. Sequential treatment of infected wounds with SSD–CSM-FPEG followed by ASC-FPEGs (SSD-CSM-ASC-FPEG) significantly reduced bacterial colonization (9 log reduction) and pro-inflammatory cytokine (TNF-α) expression. A significant increase in neovascularization markers; NG2 and vWF was also observed. Histological analysis indicated the wounds treated with SSD-CSM-ASC-FPEG increased amount of dermal collagen matrix deposition, a thicker granulation tissue on day 21 and more mature collagen on day 28. This work demonstrates that the sequential treatment of infected burn wounds with SSD-CSM-FPEG followed by ASC-FPEG reduces bacterial infection as well as promotes neo-vascularization with improved matrix remodeling.

## Introduction

Burn wound infection can potentially lead to severe morbidity and can have a major health-economic burden [1–3]. Thermal injuries are prevalent in both civilian and military population and constitute approximately 5 to 10% of all warfare military casualties [4] and are among the most expensive non-fatal injuries to treat and account for a substantial economic loss for society. Each year over 300,000 people die worldwide, and about 90% of burns occur in countries with low and middle incomes (WHO 2008 world report on prevention of childhood injuries). Morbidity after large burns is often considerable and commonly associated with reduced quality of life. Burn injuries are often complicated by infection which results in long rehabilitation and hospitalization time [2,5]. Infection requires immediate treatment to reduce the possibility of secondary complications and to prevent impairment of the healing process. The most prevalent infection are caused by the pathogenic microbes *Pseudomonas aeruginosa* and *Staphylococcus aureus* [6]. Thermal injury induces an immune compromised state; infection further exacerbates the normally occurring array of events such as resolution of inflammation, epidermal maturation and neo-vascularization [1,7–9]. In addition, burn wound eschar often promotes bacterial colonization and imparts a considerable reduction in oxygen tension, followed by delayed re-epithelialization, and wound closure [10,11]. Mitigation of infection is therefore crucial for a wound to follow a normal healing pattern.

Silver sulfadiazine (SSD) is one of the best identified topical antibacterial agents to control wound infection [12,13]. SSD possesses a broad spectrum of activity against gram-positive and gram-negative bacteria as well as fungi [14–16]. The ability of SSD to reduce early invasive wound sepsis at low concentration has made it a drug of choice for burn wound injuries [17]. However, usage of silver based creams in large burn wounds results in toxicity due to systemic absorption of silver ions and currently available formulations lack the ability to control the release of SSD to prevent serum silver concentration to reach toxic levels [18–21]. To address this problem, we developed a controlled release formulation for delivering SSD from chitosan microspheres impregnated in polyethylene glycol (PEGylated) fibrin gels (SSD-CSM-FPEG) [22]. Chitosan has the natural ability to interact with host cells and have been previously used for the controlled release of drugs [23]. Entrapment of SSD in CSM resulted in a reduced burst release of SSD. The release of drug followed a first order kinetics and maintained a controlled release state for 72 h. Further, drug released from the fibrin hydrogel exhibited microbicidal activity against *Staphylococcus aureus* and *Pseudomonas aeruginosa*[22].

Most antimicrobial wound dressings are primarily designed with a view to prevent the recurrence of infection, with less focus on the quality of healing [24]. In addition to providing an antimicrobial cover, it is also desirable that the dressing provides a viable environment for the host cells to granulate. In this respect, the use of PEGylated fibrin is optimal since it possesses an inherent ability to act as a three-dimensional provisional matrix. This offers a viable environment for host cells to granulate and facilitates burn wound regeneration. The hydrogel degrades over time, subsequently promoting new granulation tissue structure.

Of note, recruitment of mesenchymal stem cells (MSCs) to the wound site is currently thought to be a crucial early event in the tissue regeneration process [25]. In particular, adipose derived stem cells (ASCs) can modulate the skin wound healing process by inducing faster re-epithelialization, fibroblast activation, migration, proliferation and collagen synthesis. We have also previously demonstrated *in vitro* that ASCs embedded in PEGylated fibrin hydrogel, themselves formed tube-like structures[26] [22]. ASCs within the hydrogel exhibits phenotypic characteristics of pericytes (Neural/glial antigen 2/chondroitin sulfate proteoglycan (NG2^+^) and platelet derived growth factor receptor beta (PDGFRβ^+^)) [22], which are vital for further endothelial cell infiltration during neo-vascularization [27–29]. We have also published data on the feasibility and efficacy of ASCs delivery into a burn wound in a porcine model [30]. Therefore, following the conclusion from our previous paper, in this infected model (current work), once the SSD helps to mitigate the infection, the ASCs seem to improve neo-vascularization corroborating to our previously published results.

Conceivably, SSD-CSM-FPEG stands out to be a potent wound dressing with both angiogenic and antimicrobial activity. However, further evaluation of the efficacy of SSD-CSM-FPEG, replicating *in vitro* results in an animal skin injury model is highly warranted [22]. Based on these premises, the major objective of this study is therefore geared towards evaluating the efficacy rendered by a sequential treatment of SSD-CSM–FPEG followed by ASC-FPEG (SSD-CSM-ASC-FPEG) for the treatment of infected burn wounds using a preclinical rat model.

## Results

### Treatment with SSD-CSM-FPEG reduces bacterial infection

**Fig 1:**
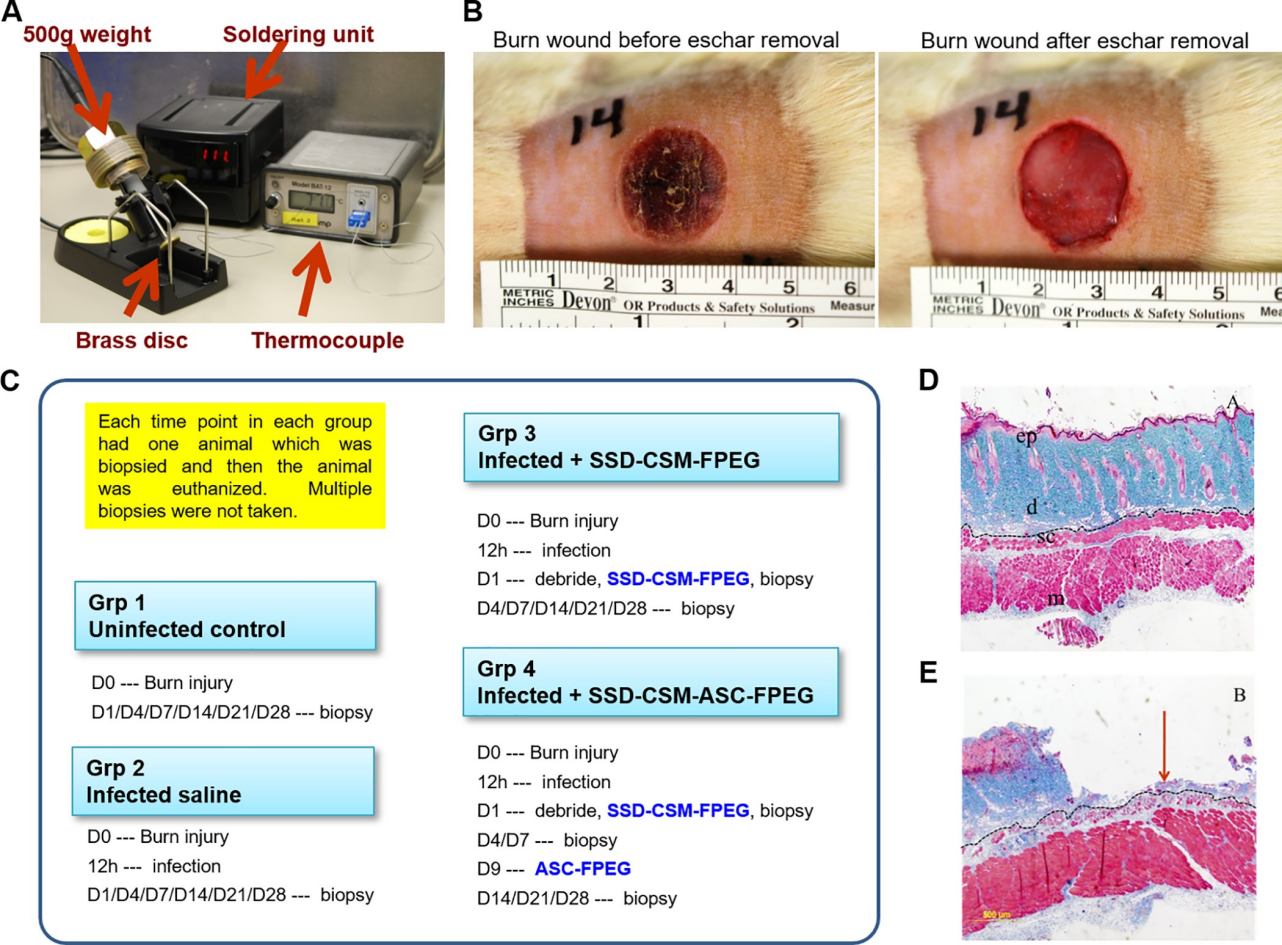
A) Burn device, B) Photographic image of the burn wound, C) Timeline of experiment, D) Histology: unburnt skin—All layers of skin is intact and viable E) Histology: 10s burn–Epidermis and most of dermis is lost (partial thickness burn).

**Fig 2:**
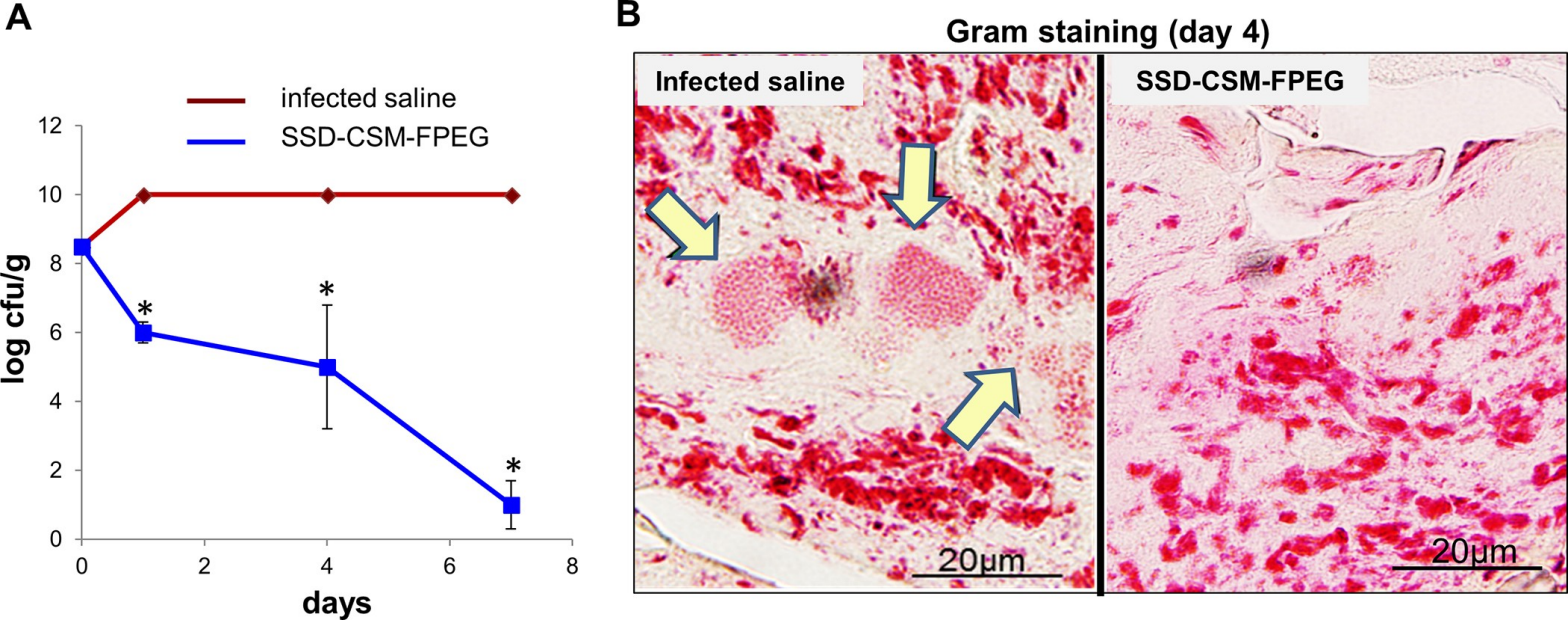
SSD-CSM-FPEG reduces bacterial load. (A) Bacterial growth kinetics upon treatment with SSD-CSM-FPEG. (B) Gram staining demonstrating presence of bacteria (indicated by arrows) in infected saline samples while absent in SSD-CSM-FPEG treated samples. (*n* = 4, *p*<0.05).

A deep partial thickness burn was created in anaesthetized rat’s dorsum (Fig 1) and infected with a challenging inoculum of 10^8^ cfu/ml *P*. *aeruginosa*. The efficiency of SSD-CSM–FPEG against microbial challenge was assessed both quantitatively (log cfu/g) and qualitatively (gram staining). The SSD-CSM-FPEG treated rats started to show a slight decrease in microbial count from initial inoculums (from 10^8^ cfu/g to ~10^6^ cfu /g) within 24 hours (Fig 2A). On day 4, a significant decrease in infection (~10^5^ cfu/g; *p* <0.05) was observed. Day 7 analysis of biopsies showed bactericidal effect of treatment with a significant decrease (< 100 cfu/g; *p*<0.05) in microbial load from initial inoculums compared to the control groups, which still showed a very high load of bacteria on the wound bed (>10^9^ cfu/g) beyond the range of detection. Gram staining further confirmed the presence of bacteria in the control samples but none in the SSD-CSM-FPEG treated samples (Fig 2B). (*n = 4)*.

### SSD-CSM-FPEG treatment suppressed inflammation

**Fig 3:**
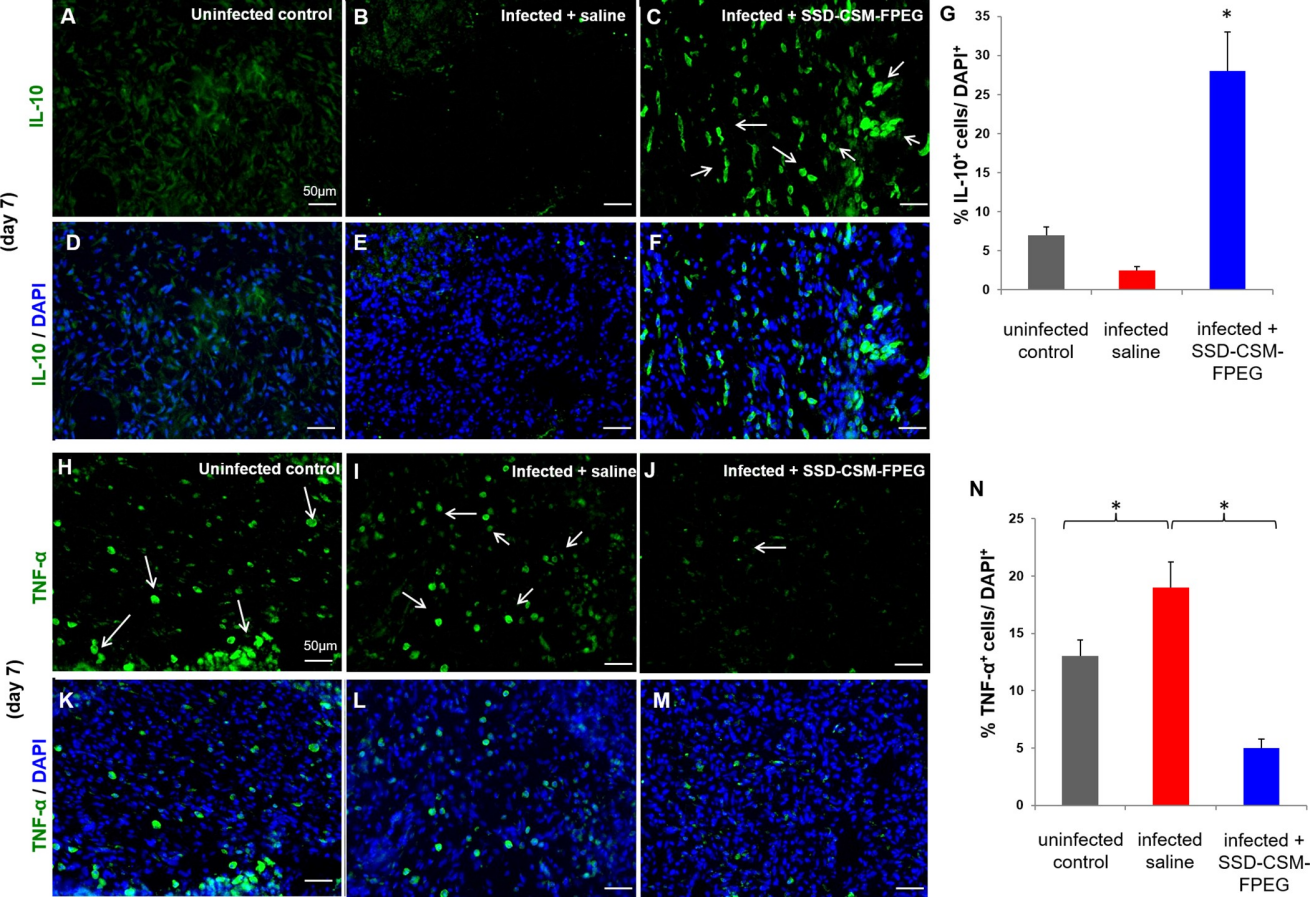
SSD-CSM-FPEG reduces inflammation. (A-G) SSD-CSM-FPEG exhibits increased anti-inflammatory marker IL-10. Day 7 wound tissue exhibits increased expression of IL-10 (green, white arrows) in wound tissue treated with SSD-CSM-FPEG as compared to infected saline treated samples. (*n* = 4, *p*<0.05). (H-N) SSD-CSM-FPEG exhibits decreased pro-inflammatory marker TNF-α. Day 7 wound tissue exhibits decreased expression of TNF-α (green, white arrows) in wound tissue treated with SSD-CSM-FPEG as compared to infected saline treated samples. (*n* = 4, *p*<0.05).

Immunofluorescence staining was performed to determine the inflammatory status of the infected wounds after treatment with SSD-CSM-FPEG. Biopsies of the tissue sections collected from uninfected control (Fig 3A and 3D), infected saline (Fig 3B and 3E) and SSD-CSM-FPEG treated infected rats (Fig 3C and 3F) were stained with anti-inflammatory cytokine antibody IL-10. Clearly, a distinct expression of IL-10 was observed in the group of rats treated with SSD-CSM-FPEG and further confirmed through quantitative measurement of relative fluorescence intensity; a significant change in percentage fluorescence was measured in the treatment groups compared to the uninfected controls and infected saline (Fig 3G). Corroborating to our results, other groups have similarly reported that silver by itself can exert an anti-inflammatory effect. A recent in vitro study shows potent inhibition of key inflammatory cytokines, viz., IL-1β, IL-6 and TNF-α at concentrations ranging from 10–20 μg/mg, which is much lesser that the toxic range. In addition, silver nanoparticles strongly inhibited production of other cytokines like INF-γ, IL- 8, IL-11[31–33]. Hence this observation can be attributed to the presence of silver in addition to the reduction of the infection stimulus. Furthermore, tissue sections of uninfected control (Fig 3H and 3K) and infected saline samples (Fig 3I–3L) were noted to be stained positive for the pro-inflammatory cytokine marker TNF-α. In contrast, SSD-CSM-FPEG treated group exhibited low to negligible TNF-α positive signal (Fig 3J and 3M). Of note, quantitative analysis further confirmed our observations with infected control exhibiting significantly high level of TNF-α in comparison to SSD-CSM-FPEG treated group (Fig 3N).

### PEGylated fibrin hydrogel with ASCs facilitates neo-vascularization

**Fig 4:**
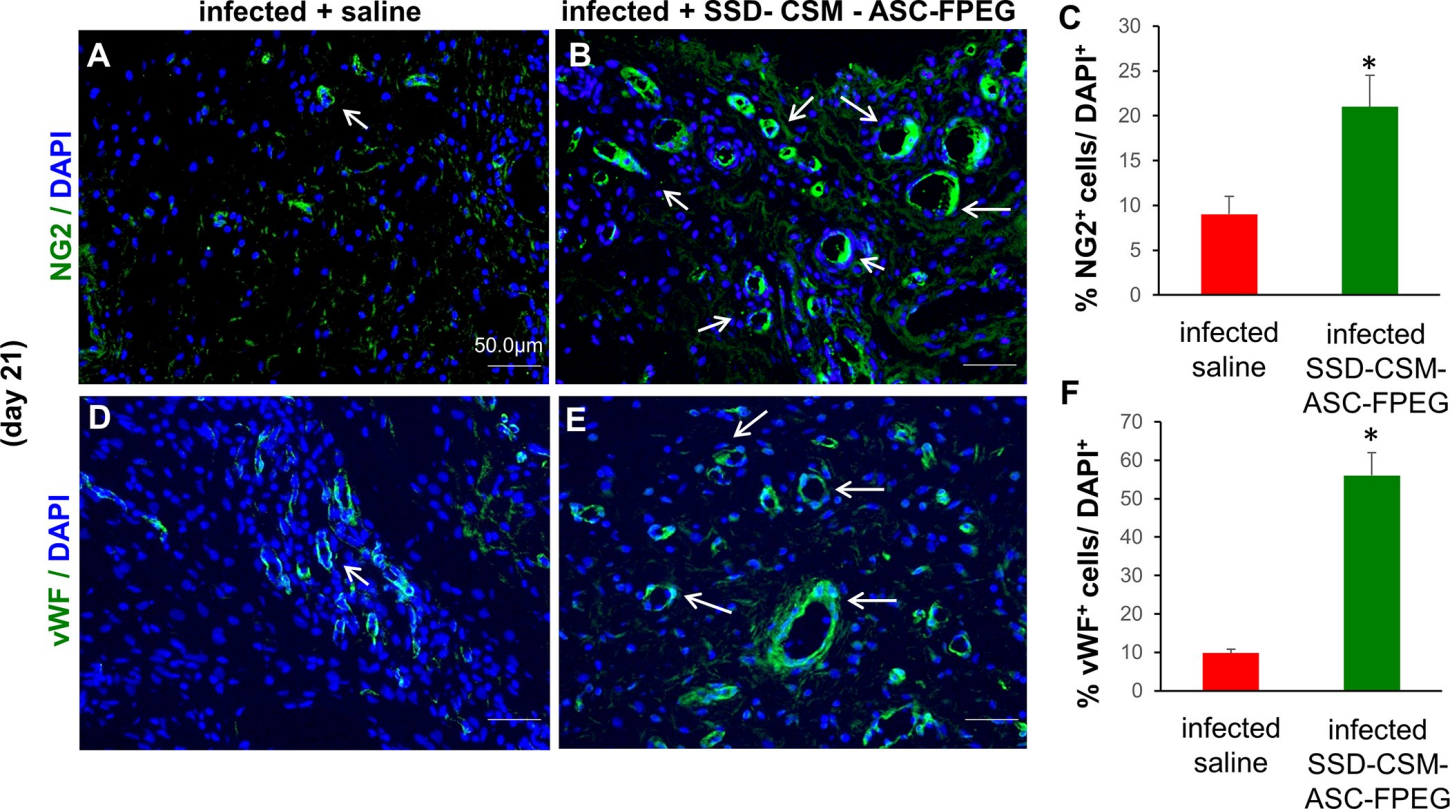
SSD-CSM-ASC-FPEG facilitates neo-vascularization. Day 21 SSD-CSM-ASC-FPEG treated samples exhibit A-C) pericyte marker NG2 expression, indicating neo-vascularization, and D-F) endothelial cell marker von-Willebrand factor (vWF) expression. These markers were not expressed in the infected saline treated samples (*n* = 4, *p*<0.05).

Prior reports show interleukin 10 (IL-10) to be one of nidus for neovascularization, i.e., formation of new blood vessel [34,35]. Since we observed increased level of IL-10 expression in the SSD-CSM-FPEG groups, the tissue sections collected on days 7, 14 and 21 were stained for angiogenic markers using primary antibodies specific to neural/glial antigen 2 (NG2, Fig 4A–4C) and von-Willebrand factor (vWF, Fig 4D–4F). Precisely, NG2 is a trans membrane proteoglycan on nascent pericytes with a functional role in neovascularization, responsible for the formation of patent blood vessel [36]. A significant increase in NG2 expression was observed in the day 21 wounds treated with SSD-CSM-ASC-FPEG in comparison to untreated infected wounds. Positive expression of NG2 represents formation of stable vasculature. Along with NG2, a concurrent increase in von-Willebrand factor (vWf; a glycoprotein produced uniquely by endothelial cells and is a marker for objective assessment for tissue angiogenesis [37]) was also found in the treated tissue. In parallel, an increase in vascularity, measured by microscopic counting of vessels stained for the endothelial cell–specific protein von Willebrand factor, was observed in the SSD-CSM-ASC-FPEG treated groups.

### PEGylated fibrin hydrogel with SSD and ASCs treatment improves wound granulation

**Fig 5:**
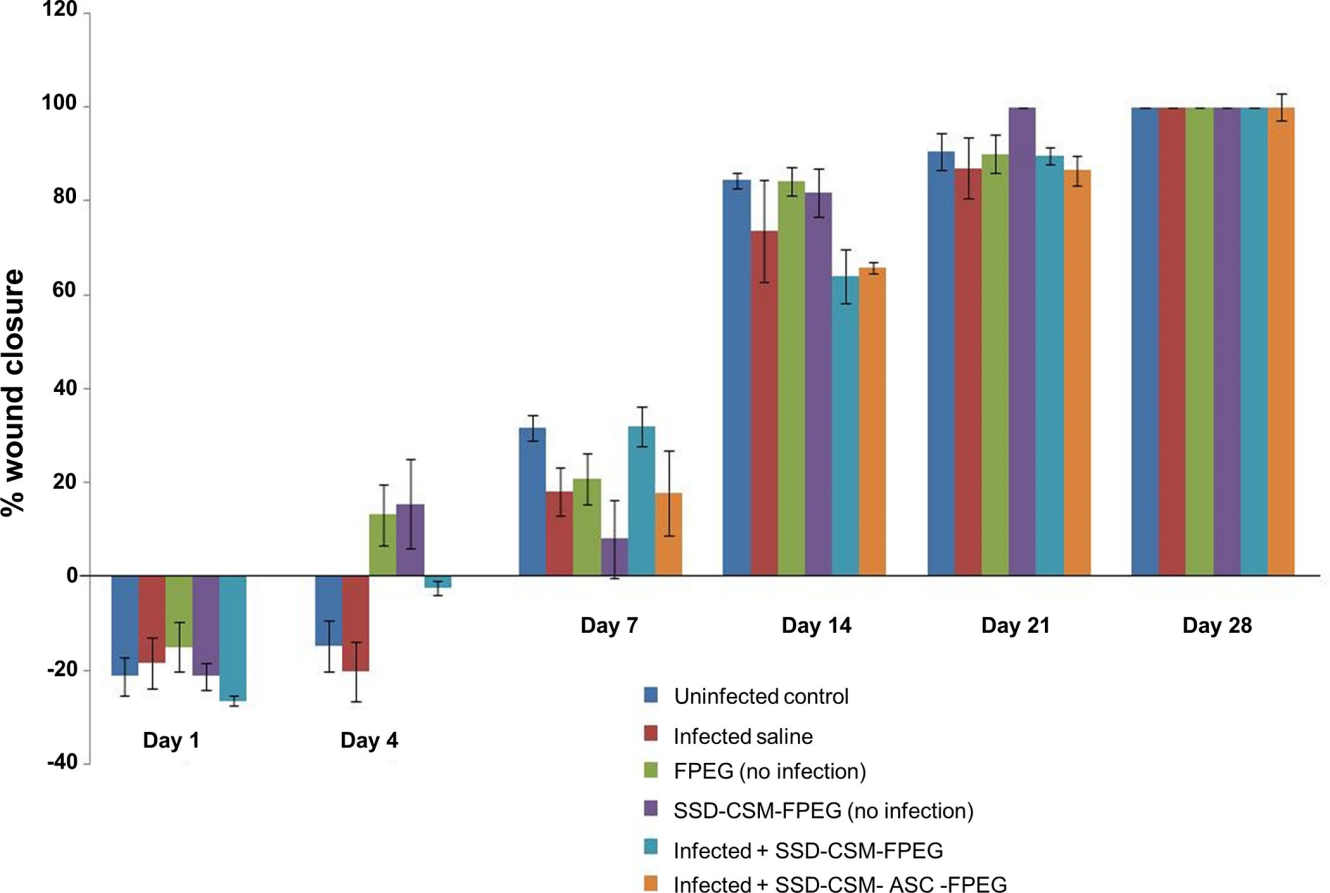
Percentage wound closure observed over 28 days in infected/ non-infected groups treated with SSDM-CSM-FPEG with or without ASCs. (*n* = 4).

The wound closure pattern of infected wounds treated with the synergistic treatment of SSD–CSM-FPEG with or without ASCs was assessed by measuring wound closure rate and by Mason’s trichrome staining and pentachrome staining. There were no significant differences in the rate of wound closure between groups. A delayed healing response was observed during initial days. However, by day 7, a minimal healing rate changes between groups was observed; which coincides with reduction in bacterial load in the infected wounds treated with SSD-CSM-FPEG (Fig 5). After 21 days post burn, wound in all the groups approached 90% closure irrespective of infection status and by 28 days, all the groups showed complete wound closure.

**Fig 6:**
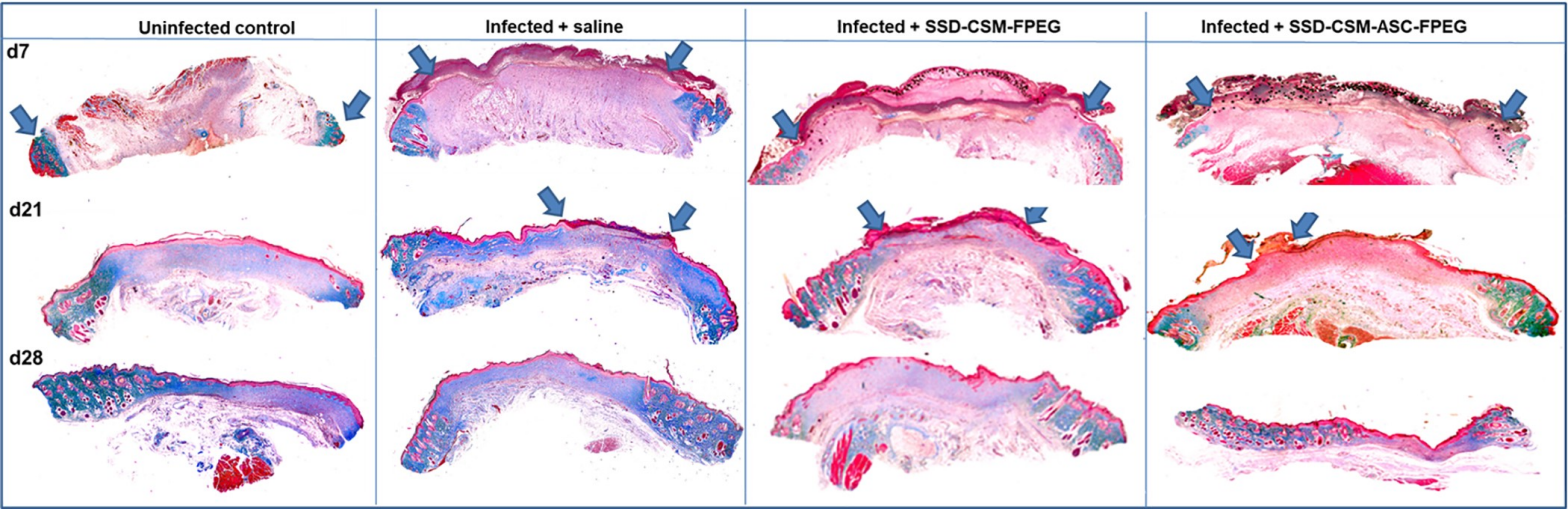
Wound closure characteristics. Representative Masons trichrome showing wound histology on day 7, day 21 and day 28. Interpretation of Masons trichrome staining is as follows: red: keratin and muscle fibers; blue or green: collagen and bone; light red or pink: cytoplasm; and dark brown to black: cell nuclei. SSD-CSM-ASC-FPEG treated samples was observed to have a significantly thicker granulation tissue (*n* = 4).

**Fig 7:**
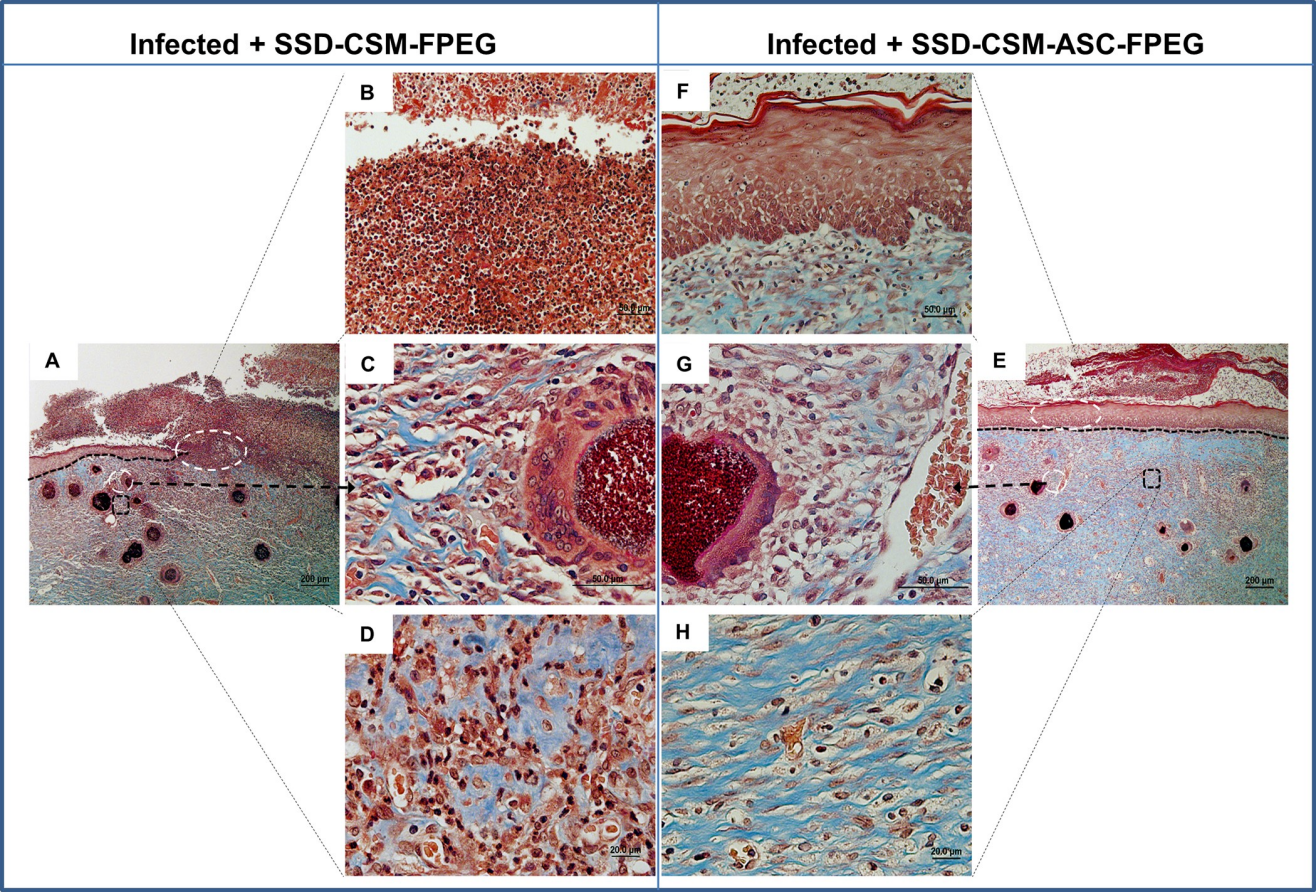
Masson’s trichrome stained tissue sections of wounds treated with SSDM-CSM-FPEG without (A-D) or with ASCs (F-E) on day 14. B-D are zoomed in views from A and F-H are zoomed in areas from E.

**Fig 8:**
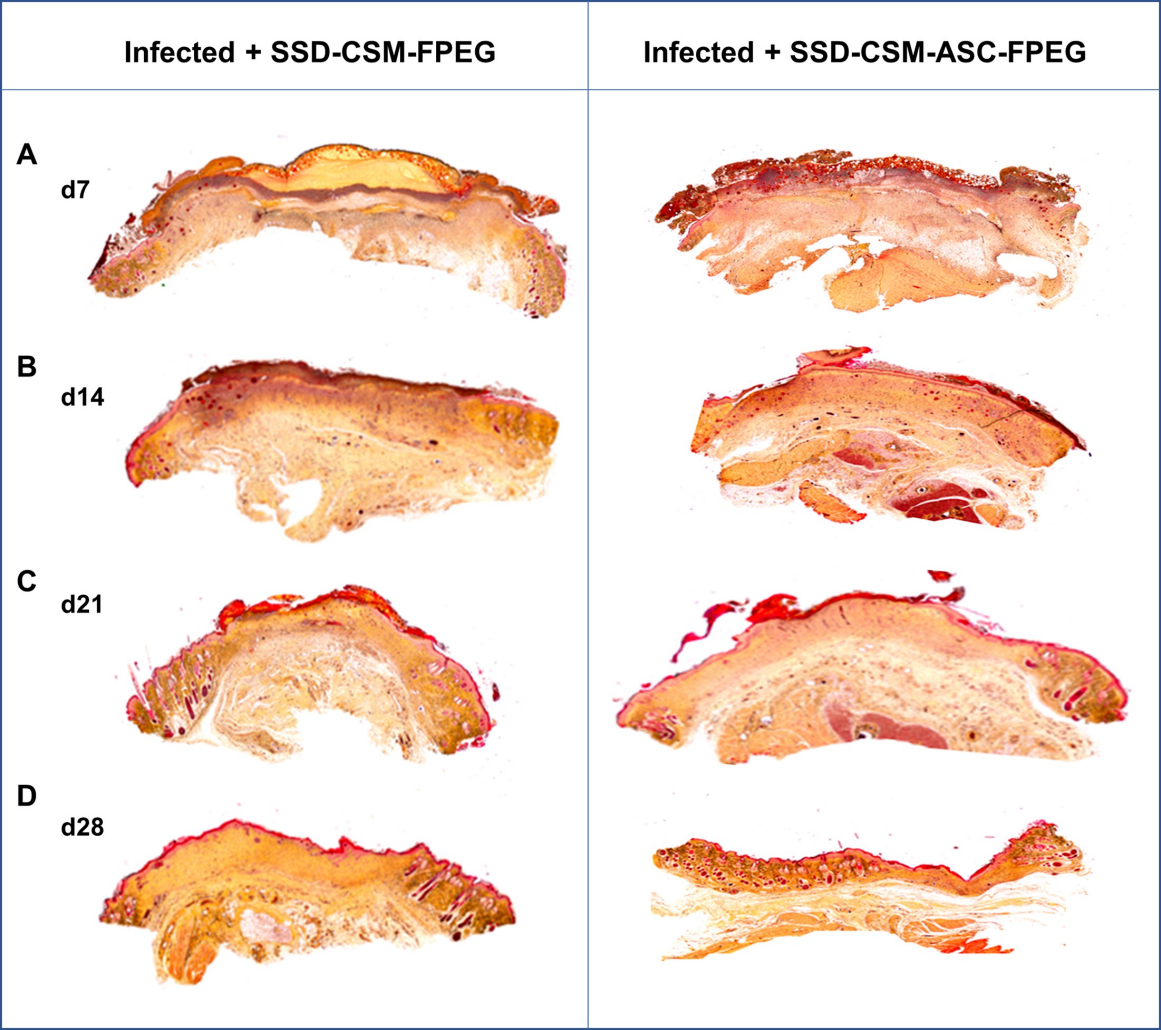
Wound closure characteristics. Representative Movatt’s pentachrome staining showing wound histology on (A) day 7, (B) day 14 (C) day 21 and (D) day 28. Interpretation of Pentachrome staining is as follows: blue: ground substance, mucin; bright red: fibrin; red: muscle; yellow: collagen and reticular fibers; black: cell nuclei. SSD-CSM-ASC-FPEG treated samples was observed to have a significantly thicker granulation tissue. (*n* = 4).

Masson’s trichrome stain was helpful to evaluate the collagen deposition and organization during healing (Fig 6). Both the groups treated with SSD-CSM-FPEG with and without ASCs, show no bacterial infiltration after day 7. The chitosan microspheres were clearly seen to be embedded within the wound bed and were found to be wide spread on scab region found on top of the granulating wound bed. The microspheres were still found within the re-organizing dermal wound bed on day 14. In both the groups, the surrounding tissue was minimally infiltrated with macrophages or with less inflammatory cells (Fig 7C and 7G) which indicates the controlled release of SSD treatment to be compatible in treating these wounds. The PEGylated fibrin matrix in both treatment groups was indistinguishable after day 14 and mostly replaced by host granulation tissue component. In comparison rats treated with SSD-CSM-ASC-FPEG showed increased amount of dermal collagen matrix deposition in comparison to the SSD-CSM-FPEG treatment (without ASCs) (Fig 7D and 7H). Further, the wound bed of rats treated with SSD-CSM-ASC-FPEG started to show more defined dermal-epithelial boundary with remnant of sloughing scab on top of the wound bed (Fig 7F). The rats treated with SSD-CSM-FPEG with no ASCs showed a less organized dermal layer with associated bed of unorganized granulation tissue and an absence of any re-epithelialization (Fig 7B). The neo-synthesized collagen could be clearly visualized (stained blue) in wound treated with SSD-CSM-ASC-FPEG. The collagen deposition and the embedded chitosan microspheres were also evident from the Movat’s pentachrome staining (Fig 8).

After 21 days, SSD-CSM-ASC-FPEG treatment groups showed a defined epithelial layer advancing towards the center underneath the adnexial scab layer progressing towards complete closure (S1B Fig). Whereas the SSD-CSM-FPEG treatment group still showed remodeling dermis associated with unorganized granulation tissue with significant amount of scab tissue (S1A Fig). Though the leading epidermal edge could be seen, still the distinct epithelial-dermal demarcation was not evident. A thicker granulation tissue (than the infected untreated samples) was also observed in the SSD-CSM-ASC-FPEG treated samples (S2A and S2B Fig), which eventually regressed to near normal skin (thus alleviating concerns about hypergranulation). By day 28 wounds of both the treatment groups showed complete re-epithelialization and prominent remodeling of the dermal layer. Although both the hydrogel-treated groups showed comparably similar re-epithelialization, the groups treated with SSD-CSM-ASC-FPEG showed well-organized dermal and epithelial layers with a significantly better re-epithelializing wound margin and minimal scab adhering to the wound bed. In addition, the dermal layer of rats treated with SSD-CSM-FPEG were still undergoing evident remodeling along with associated epithelial layer.

**Fig 9:**
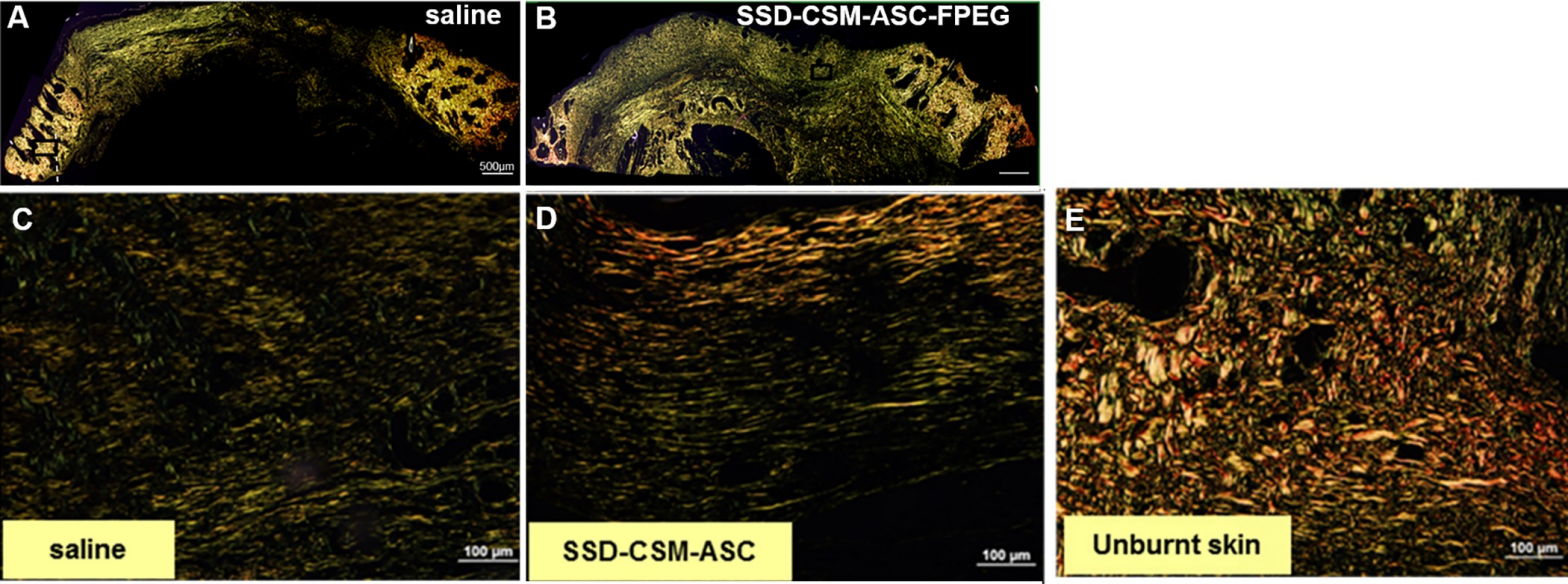
Maturation of collagen. Picrosirius staining on day 28 infected burn wounds treated with (A) saline or (B) SSD-CSM-ASC-FPEG. Zoomed images from A shows (C) pre-dominantly thinner (green) regrowing fibers in infected saline control samples, (D) more number of thicker, arranged mature collagen fibers (red) in wounds treated with SSD-CSM-ASC-FPEG, and a basket like mature collagen in the (E) unburnt skin.

Picrosirius red (PSR) stain showed matured collagen turn over (collagen 1: collagen 3) with wounds trending to close with a collagen distribution (2.8:1) similar to that of unburned controls (2:1) (Fig 9A–9E; *n = 4)*. By day 28, the collagen has matured into thicker yellow and organized bundles in the treated tissue, while the control samples were still found to have predominantly re-growing thinner unorganized collagen fibers. It is worth mentioning that Herovici stain is generally used to differentiate young and mature collagen and this stain colors mature dense collagen red while newly formed collagen are colored blue [38–40]). Herovici staining on day 28 samples (remodeling phase) further confirmed the formation of organized mature collagen in the wounds treated with SSD–CSM-ASC-FPEG (S2C and S2D Fig). This observation along with increase in neovascularization markers is consistent with reports of increased granulation tissue supporting new vessel formation [41–43].

## Discussion

During acute healing process, infiltration of inflammatory cells ceases within a few days after injury, whereas, in infected wounds a persistent pro-inflammatory and reduced anti-inflammatory cytokines levels are reported. With chronic inflammation, the mechanical stability of the provisional matrix is diminished hindering the development of new blood vessels [44]. Transition of the inflammatory phase to the proliferative phase and the formation of granulation tissue is also considerably slowed [45]. Increased levels of endopeptidases, mainly the matrix metalloproteinases (MMPs), result in an imbalance in matrix synthesis and matrix degradation leading to delayed healing [46]. However, antibiotic treatment approaches applied to infected burn wounds are primarily targeted to reduce the bacterial colonization with less of an impact on the subsequent healing phases/processes. Hence, therapeutic intervention to simultaneously control infection and improve the progress of organized healing phases would be an advancement over currently available strategies.

Selection of a carrier system for on-site delivery of antimicrobial drug at a desired rate of release is an important consideration to prevent burn wound infection. SSD is the widely accepted, FDA approved, topical agent to control bacterial colonization of burn wounds [12,47–49]. However, silver is toxic to the cells [18–21,50] and clinical studies have shown that in burned patients (with >5% total body surface area) topical application of SSD can result in approximately twentyfold increase of blood silver levels [51,52]. In general, a burn wound represents an open compartment system and it is extremely difficult to tailor a ‘defined-dosage drug release system’. Application of an antibiotic wound dressing often results in undesired high SSD dosage exposure. Especially, in cases of second degree partial thickness wound, will result in excessive silver absorption through peripheral blood vessels present in the wound bed. There are evidence that show silver binds to serum proteins and gets absorbed into systemic circulation [19]. Therefore, a major factor that was considered, was to control the release kinetics of SSD at the infected wound site for a prolong period.

Chitosan is a natural glycosaminoglycan polymer with excellent biocompatibility, cost effective and is available in bulk [53,54]. Chitosan microspheres loaded with SSD used in this work were developed via a novel water-in-oil emulsion technique and are porous with needle-like structures evenly distributed over the spheres [22]. In our earlier study, we designed the fibrin hydrogel based wound dressing based on challenging doses of initial bacterial inoculum (10^8^ cfu/mL) and SSD was loaded into the chitosan microcarrier to prevent such a challenging bacterial load [22]. Both SSD–CSM and SSD–CSM–PEGylated fibrin gels were able to maintain an active antibacterial concentration of SSD. In other words, we could control the rate of release at minimal inhibitory concentration (MIC) levels, the ‘desired release concentration’, at the wound site for prolonged period of time without the need to change or apply another wound dressing.

As reported previously, *in vitro* drug release studies have shown that a burst release of 27.02% in 6h was achieved followed by a controlled release over 72 h with an equilibrium concentration of 27.7%; and a microbicidal activity at 125 and 100 μg/ml was exhibited against *Staphylococcus aureus* and *Pseudomonas aeruginosa*, respectively [22]. The optimized release kinetics for the SSD-CSM and SSD–CSM–FPEG hydrogels and the MIC against P. aeruginosa was observed to be 9 mg (90 ± 1.0 μg/ml SSD) and the MBC was 10 mg (100 ± 0.25 μg/ml SSD) [22].

In our recent *in vivo* study, using infected porcine full thickness excision wound model, we have shown SSD delivery from CSM using PEGylated fibrinogen to significantly reduce the infection than the standard of care silvadene cream [55]. Additionally, fibrin hydrogels were chosen for delivery of the SSD loaded chitosan microspheres to the wound because FPEG is a three-dimensional hydrogel scaffold and act as a provisional matrix; wherein the hydrogel degrades over time, subsequently promoting new granulation tissue structure and new blood vessel to form [26]. Moreover in our latest study we have shown PEGylated fibrin based hydrogel applied to a debrided porcine burn wound exhibit minimal inflammation and foreign body reaction, modulate inflammation and improved wound healing [56–58]. Our current treatment module with SSD–CSM-FPEG resulted in a decrease of pro-inflammatory cytokine TNF-α and increase in anti-inflammatory cytokine IL-10, indicating a resolution of inflammation following subsidence of infection. We analyzed inflammatory status on day 7 as it can be correlated to the time where the infection completely subsided. Our results corroborates to the earlier findings of an increase in IL-10 expression leading to decrease in the inflammatory response following an injury [59,60]. Therefore, the current treatment protocol creates an environment conducive for regenerative wound healing.

Another desirable feature in the treatment approach will be to use a dressing that can promote revascularization alongside reducing infection and inflammation. Various agents have been developed to control bacterial infection in burn patients [61], but none appear to promote revascularization. Revascularization is particularly important for burn wound healing because in severely burned tissues, the cell and vasculature are often destroyed. Successful wound healing proceeds through an orderly progression of phases that relies upon a vasculature by which cells and soluble factors can reach the wounded tissues and depends on the development of conditions that closely mimic the *in vivo* microenvironment [62]. Recently, stem cells have been used for wound healing and tissue regeneration [63]. Adult mesenchymal stem cells (MSCs) are a population of fibroblast-like progenitor cells that possess the capacity for self-renewal, long-term viability and multiple lineage potential [64,65]. MSCs have been isolated from several tissue types including cartilage, muscle, systemic blood, umbilical cord blood and vasculature, skin, and adipose tissue [63,66–71]. Clinically, adipose tissue presents a large resource for MSCs and can be isolated in large quantities with minimally invasive technique [71,72]. Control of infection and proper preparation of the wound bed are of vital importance to success of stem cell based therapies [73] and therefore, a combination of infection mitigation followed by stem cell delivery can improve efficacy of the treatment. MSCs themselves provide a significant advantage in reducing excess inflammation from any contaminants in the wound during injury and treatment [74]. Although, in the current study we have delivered SSD followed by ASCs, our previous data show SSD did not affect the proliferation of ASCs in the same fibrin delivery scaffold, with no change in cell viability.

Delivery of ASCs (day 9) through PEGylated fibrin hydrogel after the subsidence of infection significantly increased granulation tissue formation (day 21). Of note, the SSD-CSM-FPEG trapped within the wound bed were compatible and did not trigger exaggerated foreign body inflammatory reaction; this histological observation corroborated well with the reduced expression of pro-inflammatory cytokine in the SSD–CSM-ASC-FPEG treated rats. Newly formed collagen [75], predominantly type III in rats [76], was observed in both PSR and Herovici stained slides. Earlier studies have demonstrated that full thickness wounds in rat and rabbit models treated with ASCs results in a thicker granulation tissue, increased collagen deposition, more regular alignment of fibers and greater neovascularization [77–79]. Neovascularization or angiogenesis involves the growth of new capillaries to form granulation tissue, which acts as a matrix for proliferating blood vessels, migrating fibroblasts and new collagen[80]. Impaired granulation is a hallmark of non-healing wounds as is also observed in our infected burn wounds. Therefore, consistent with the knowledge that increased granulation tissue supports greater number of blood vessels in the dermis [41–43], we found an increase in granulation tissue in our SSD-CSM-ASC-FPEG over the saline infected samples and a significant increase in pericyte and neovascularization markers (NG2, vWF). Another striking observation is presence of larger vessels in the SSD-CSM-ASC-FPEG treated group which may suggest that these blood vessels were formed early and had more time to mature suggesting enhanced neo-angiogenesis. However, more extensive time-course experiments are needed to further investigate this observation[81]. As the granulation tissue matures, thicker, more organized collagen fibers and increased blood vessels density becomes the hallmark of a healing wound [82–88]. We noticed that although the treated samples showed increased granulation on day 21, it had remodeled by day 28, thus alleviating the concern about hyper granulation and scarring. This is also consistent with our Picrosirius staining results on day 28 wound biopsies, showing mature thick (yellow) collagen in the treated wounds as compared to thin re-growing fibers (green) in the untreated wound. We recognize that our current model is not optimal for studying wound closure, as rat is a loose skinned animal and the presence of a subcutaneous panniculus carnosus muscle in rats, contributes to healing by contraction[89,90]. Unrestrained rapid contraction counteracts the accumulation of granulation tissue, and therefore does not accurately reflect human wound healing by secondary intention. Hence, in the current study, although we do not see a significant difference in wound closure between the ASC treated vs the untreated group, an increased granulation tissue and better vasculature may be more accurate predictors of quality of healing and prevention of wound recurrence or surgical dehiscence.

A limitation of this study is the sample size. Since, we used each rat for a single time point and did not do multiple time-points on a single rat, although the biological replicates for each group was n = 4, for all the time points together (day 1, 4, 7, 14, 21, 28) we used 96 rats for this work (4 groups X 6-time points X 4 rats/ time point). While, recognizing that increasing the number of animals could have possibly increased the significance of the results, it would also have significantly increased the total number of rats of the study resulting in logistical issues of handling such large number of animals.

Nonetheless, the overarching goal for this work was to do a screening study in a rodent model and then extend the findings to a pre-clinical porcine model to examine skin graft take. As a preliminary study, the antimicrobial efficacy of SSD-CSM-FPEG has been evaluated by our group using a full thickness porcine wound infected with Pseudomonas aeruginosa [91]. Additionally, we have demonstrated the feasibility and efficacy of delivery of allogeneic ASCs in FPEG as an adjunct to meshed autografts and have shown that contraction seen after meshing of graft can be prevented by FPEG hydrogels [30]. The effectiveness of the ASCs in improving graft take in an infected wound has not been studied yet and we have planned to perform such experiments in future. The results revealed from this study also qualify for more in-depth experiments to determine long term outcomes such as contraction and scarring. Taken together, our studies underscore the development of new technologies with the objective of combining antimicrobials/antibiotics, biomaterial scaffolds, and stem cells that will reduce infection and as well as enable the active healing.

## Materials and methods

### Rat burn wound model

This study has been conducted in compliance with the Animal Welfare Act, the implementing Animal Welfare Regulations, and the principles of the Guide for the Care and Use of Laboratory Animals. All animals received laboratory grade commercial feed and water *ad libitum*. The animals were anaesthetized using a vaporizer setting of 1–3% isoflurane delivered with a nose cone on a Bain circuit hooked to the rodent gas anesthesia machine, and the hair on the dorsum was shaved. A circular-shaped burn injury was created by heating a brass soldering device to 87°C by a thermocouple probe and thermometer (Model BAT-12, Physitemp) capable of monitoring real-time temperature in direct contact with the brass disk (17 mm diameter and 2.5 mm thickness). The heating brass plate was loaded on the dorsal area of rats for 10 seconds with a constant force using 500g of weight equipped around the soldering tip (Fig 1). After 12hrs, the depth of the wound was histologically assessed by staining the skin biopsy (5–7μm thick sections) and representative images are shown with Masson’s trichrome staining (Fig 6). The biopsies were collected to include the entire wound with a 2mm circumference of surrounding normal skin. From that, first 2–3 biopsies (4mm) were randomly taken within the wound bed for microbiology purpose. Then a strip spanning from right to the left was taken from the un-biopsied area and used for histology sectioning.

### Induction of infection

*Pseudomonas aeruginosa* (ATCC #15442, Manassas, VA), culture was prepared by inoculating five colonies from a freshly cultured plate into 10mL of Muller Hinton Broth (MHB), (Becton–Dickinson, Franklin Lakes, NJ) and incubated at 37°C until an OD_600_ of 0.1 (approximately 3–6 hrs). From this culture 100μL was transferred to 10mL of fresh MHB and incubated to attain exponential phase as measured by an absorbance of OD 0.1 at a wavelength of 600nm (approximately 3hrs, 10^8^ colony forming units (cfu)/mL). Appropriate dilutions were made to prepare bacterial challenge inoculum of 10^8^cfu/ml of saline for inducing infection.

The 17mm partial thickness contact burn injury was placed on the dorsum of the rat (*n* = 4) as described above (Day 0). Twelve hours after injury the wound was infected with *P*.*aeruginosa*. The bacterial suspension (100μl) was carefully administered underneath the eschar, between the subcutaneous skin and paraspinus muscular layer, and left overnight (12–14 hours) to establish the infection. Positive bacterial colonization was confirmed by taking wound biopsies (0.25-1cm^2^) of depth up to the panniculus adiposus (a layer of fat underlying dermis), 12–14 hours post infection, eschar was carefully removed by releasing/dissection the dead tissue and cut along the circumference without disturbing the wound bed as well the normal unburned skin, and underlying wound tissue was carefully excised, weighed, and minced with 1mL of sterile saline. All the samples were serially diluted 1–10 times and the number of CFUs determined by plating on Muller Hinton Agar (Becton–Dickinson, Franklin Lakes, NJ).

### Bacteria quantification

To assess bacterial load in the burn wounds, biopsies were collected at different time points (Day 0 post infection, days 1, 4, 7 respectively). Wound tissue was weighed, homogenized in a known 1mL of saline, serially diluted and plated on *Pseudomonas*-selective agar (Neogen, Lansing, MI) to perform bacteria quantification. The bacterial quantification was carried out through serial dilution and plating method. In this protocol, 6 serial dilution were carried out. According to clinical lab standard, the readable cfu/plate is 100–200 colonies per dilution. Plates were incubated for 18–24 h at 37°C prior to counting viable cfu. Data from bacterial quantification are then presented as the log cfu per gram of tissue. In our study, the control group exhibited highly saturated colonization and were out of range. Therefore, we are unable to delineate discrete bacterial colonies to to provide a statistical analysis for the control group.

### Experimental timeline

A 17mm contact burn injury was created on the dorsum of anaesthetized rats and randomized into 4 groups (*n* = 4).

Group 1 (*n* = 4): ‘**Uninfected controls’**; biopsy at D1/4/7/14/21/28
Group 2 (*n* = 4): ‘**Infected saline’** Infected with *P*.*aeruginosa* 12 h after burn, debrided at D1 after burn, treated with saline and biopsied on day 1/4/7/14/21/28. Saline was applied topically (100μl) spread over the wound bed and continued with a secondary cover.
Group 3 (*n* = 4 for each time point): ‘**Infected + SSD-CSM-FPEG’** Infected with *P*.*aeruginosa* 12 h after burn, debrided at 24 h after burn, treated with SSD-CSM-FPEG on day 1 and biopsied on day 1/4/7/14/21/28 after burn.
Group 4 (*n* = 4 for each time point): ‘**Infected + SSD-CSM-ASC-FPEG’** Infected with *P*.*aeruginosa* 12 h after burn, debrided at 24 h after burn, treated with SSD-CSM-FPEG followed by treatment with ASC-FPEG on day 9 after burn, and biopsied on day 14/21/28.

All the rats, following burn (± infection) were debrided on day 1 to remove the eschar and after applying treatments were covered with aquacel hydrocolloid semi-occlusive bandage that retains moisture (Convatec, Greensboro, NC). The secondary dressing was changed after every 3 days. Wounds were cleaned with saline and dressing was changed. Tissue samples were collected on designated biopsy days and stored for further experimental analyses. As a control, FPEG and SSD-CSM-FPEG was also tested to make sure that the hydrogel and SSD has no deleterious effect on the healing. (Fig 1). Each time point in each group had one animal which was biopsied and then the animal was euthanized. Thus, each wound/rat was biopsied for only a single time point and the same burn wound was not biopsied in the subsequent time-points. (A CSM-FPEG group alone without the antimicrobial was not feasible to include because the fibrin is a protein-rich source which gets disintegrated if not combined with an antibacterial agent.)

### SSD-CSM-FPEG

PEGylated fibrin hydrogels were prepared as previously described [92,93]. Briefly, succinimidylglutarate polyethylene glycol (SG-PEG-SG, 3400 Da, NOF America Corp.) was added to fibrinogen (Sigma–Aldrich) at molar ratio of 1:10 (SG-PEG-SG: fibrinogen) in Tris-buffered saline, pH 7.8, and further incubated for 20 min at 37°C. An equal volume of thrombin (Sigma-Aldrich) in 40mM CaCl_2_ at a final concentration of 10U/mL was added and incubated for 10 min at 37°C. CSM were prepared by a water-in-oil emulsification process with simultaneous ionic coacervation using our previously published protocol [22]. Briefly, using an overhead stirrer, chitosan (3% solution in 0⋅5M acetic acid, Sigma-Aldrich, St. Louis, MO) was emulsified in an oil mixture of soya oil (Sigma-Aldrich) and n-octanol (Acros Organics, New Jersey, NY), with span 80 (Sigma-Aldrich) as an emulsifier. Micelles of chitosan were slowly solidified using 1% w/v of KOH in n-octanol. After ionically cross-linking the chitosan micelles, the oil phase of the mixture was slowly decanted, and the CSM were recovered using acetone. Discrete microspheres were obtained by sonicating at 600Hz (Vibracell, Viewsonics, Newtown, CT) for 15 minutes under a constant amplitude of 42% with an intermittent on/off pulse of 9 seconds/4 seconds. Finally, the microspheres were dried in a vacuum desiccator. SSD (Sigma-Aldrich)-loaded microspheres were prepared by following similar procedure as mentioned above, in which 10 mg of SSD was added to aqueous phase of 100 mg chitosan and sonicated before the emulsification process. This resulted in the formation of microspheres within the 125–180 μm size range and a percentage SSD entrapment of 76.50 ± 2.8%. To prepare SSD–CSM–FPEG, SSD–CSM microspheres (25 mg) were added to the SG-PEG-SG: fibrinogen mixture followed by gelation with thrombin. A 2 ml hydrogel consisting of 50 mg of SSD loaded microspheres was used for treatment.

### Fabrication of ASCs-FPEG

ASCs were isolated from the rat perirenal and epididymal adipose tissue (male, 8–10 weeks, Harlan) as previously described as previously described [69,71]. Briefly, perirenal and epididymal adipose tissue was collected and washed with sterile Hanks buffered balance solution (HBBS) containing 1% bovine serum albumin (BSA). The tissue was minced, transferred into 25mL of HBBS, and centrifuged (500×g at room temperature for 10 min). The free floating adipose tissue layer was collected and transferred to 25mL of HBSS containing 1% fetal bovine serum (FBS) and 200U/mL of collagenase type II (Sigma-Aldrich, St. Louis, MO) for 45 min at 37°C in an orbital shaker. The digested tissue was then filtered through 100 μm and 70 μm nylon mesh filter, centrifuged for 10 min at 500×g at room temperature, and washed twice with sterile HBBS. The cell pellet was re-suspended in growth media (MesenPRO RS Basal Medium), supplemented with MesenPRO RS Growth Supplement, antibiotic–antimycotic [100U/mL of penicillin G, 100 μg/mL streptomycin sulfate, and 0.25 μg/mL Amphotericin B, and 2mM L-glutamine (GIBCO, Invitrogen, Carlsbad, CA)]. Cells were cultured on T75 flasks (BD Falcon, Franklin Lakes, NJ) and maintained in a 5% CO_2_ humidified incubator at 37°C. In general, passage 2–4 ASCs were used for all experiments.

To prepare 2 mL PEGylated fibrin hydrogels with ASCs, 500 μL of human fibrinogen (40 mg/mL) solution in PBS (pH 7.8) was combined with 250 μL bifunctional succinimidyl glutarate, SG-PEG-SG, solution (8 mg/mL), immediately followed by addition of 250 μL ASCs (1 × 10^5^) and 1 mL of human thrombin solution (at a final concentration of 10 U/ml in 40 mM CaCl_2_ solution). The hydrogels (2 mL total volume) were prepared in the CellCrown (5.3 cm^2^) mold.

### Histological and immunohistochemical analysis

Aliquots of wound biopsy specimens were fixed in 10% neutral-buffered formalin, dehydrated, and embedded in paraffin. Paraffin embedded wound biopsies were sectioned (5–7 μm) followed by staining with a standard gram stain (Remel, Lenexa, KS), in accordance with the manufacturer’s instruction to identify the presence of bacteria. Briefly, crystal violet was applied onto the tissue sections for 5 min at room temperature, and excess crystal violet was removed by briefly rinsing slides under running tap water. Gram iodine mordant was applied for 2 min onto the tissue sections followed by brief washing in tap water. To remove any non-specific crystal violet staining from slides, a Gram decolorizer solvent was applied for 30 s and then the slides were quickly rinsed under running tap water until the water ran clear. Subsequently, the sections were stained with Gram Safranin for 1 min and 40 s and followed by dehydration through a series of alcohols (95–100%) to xylene and then cover slipped.

To identify the pattern of distribution of cells within the wound, sections (5–7 μm) of paraffin embedded samples were mounted onto glass slides and stained with Haematoxylin and Eosin (H&E), as well with Masson's Trichrome stains (MTS) to observe cell nuclei as dark red to black, collagen as green or blue, and the muscle/cell cytoplasm/keratin as pink to red; Movats pentachrome stain was applied to identify elastin and fibrin along with collagen, cells and nuclei and Picro Sirius Red (PSR) and Herovici stain were used to visualize mature / immature collagen and granulation tissue respectively. Polarized images of PSR stained slides were captured to calculate the ratio of mature: immature collagen, by simply measuring the ratio of red: green fluorescence intensity. All the staining was done according to the manufacturer’s protocol.

Immunohistochemistry was performed using paraffin sections (5–7μm). Following deparaffinization, tissue sections were permeabilized with 0.5% Triton X-100 for 20 minutes, washed and incubated for 1 hour with 10% normal serum, matching the species of the secondary antibody, to block non-specific antigens. Nonspecific fluorescence was evaluated using sections incubated with respective isotype controls and fluorophore-labeled secondary antibodies. Background corrections were made to eliminate any false positive observations. All the primary and secondary antibodies used in our study were diluted with species specific 2.5% normal heat inactivated serum. Anti-IL10 (Abcam, Cambridge, MA, ab34843, rabbit polyclonal, 1:100 dilution) and anti-TNF-α (Abcam, Cambridge, MA, ab9739, rabbit polyclonal, 1:200 dilution) antibodies were used to investigate inflammation. Anti-NG2 (Millipore, Temecula, CA, AB5320, rabbit polyclonal, 1:100 dilution) and anti-vWF (Millipore, Temecula, CA, AB7356, rabbit polyclonal, 1:200) antibodies were used for analysis of neo-vascularization. The nuclei were counterstained with 5 μg/mL 4′, 6-diamidino-2-phenylindole, dihydrochloride (DAPI, Life Technologies) solution. Fluorescent images were acquired using a Leica microscope (DMI 3000, Buffalo Grove, IL). Percent fluorescent area was quantified using threshold method by the application of ImageJ software (National Institutes of Health, Bethesda, MD). For counting cells positive to a particular antibody (green color), images were input into ImageJ, converted to 16-bit grayscale images. A threshold was then set to include the desired region and particles were analyzed using ImageJ. Quantification was done by calculating percentage of cells positive for the antibody normalized to DAPI+ cells/ field[94].

### Wound measurement

Digital pictures of each wound (Nikon D3000, Nikon, Melville, NY) were acquired with a four‐sided ruler. Image analysis was performed with ImageJ software (NIH, Bethesda, MD). Photographs were callibrated against the ruler and the wound edge was traced. The area inside the tracing was used to measure wound closure. Wound healing was quantified as % wound closure [(Area of original wound—Area of actual wound)/Area of original wound] / 100. Granulation tissue was measured by measuring the distance from the base of the epidermial layer to the end of the granulation tissue layer, using ImageJ.

### Statistical analysis

For all the data collected from animal studies, results are represented as mean ± SD. Comparison between two groups was tested using Student's t-test (two-tailed). *p* values < 0.05 were statistically significant. *n* indicates number of animals from control and different treatment groups.

### Ethics statement

Animal research was conducted in compliance with the Animal Welfare Act, the implementing Animal Welfare Regulations, and the principles of the Guide for the Care and Use of Laboratory Animals, National Research Council. The USAISR Institutional Animal Care and Use Committee approved all research conducted in this study. The facility where this research was conducted is fully accredited by AAALAC International.

### Euthanasia

Euthanasia was performed by research personnel while the animal was under a surgical plane of anesthesia. A commercial euthanasia solution; Fatal Plus (0.1 mL/ 450 grams body weight) was injected intravenously while the animal is under general anesthesia.

## Conclusion

In this study, we propose a novel treatment for improving vascularization in burn wounds. We developed a two-step treatment process involving a controlled time and dose release of SSD (antimicrobial) followed by adipose derived stem cell delivery, which helps to avoid silver toxicity associated with conventional topical delivery approaches and primes the wound to regenerate.

## Supporting information

S1 Fig Movatt’s pentachrome stained tissue sections of wounds treated with SSDM-CSM-FPEG without (A) or with ASCs (B) on day 21.
(TIF)

S2 Fig Herovici staining demonstrates thick granulation tissue with immature collagen in day 21 in SSD-CSM-ASC-FPEG treated wound samples (B) (known to support neovascularization) as compared to saline treated samples (A). On day 28, more mature collagen was observed in the sample treated with SSD-CSM-ASC-FPEG (C-D).
(TIF)

## Abbreviations

SSD: silver sulfadiazine
CSM: chitosan microspheres
FPEG: PEGylated fibrin gel
SSD-CSM-FPEG: silver sulfadiazizne encapsulated chitosan microspheres embedded in a PEGylated fibrin gel
ASC: Adipose stem cell
SSD-CSM-ASC-FPEG: combination treatment of silver sulfadiazizne encapsulated chitosan microspheres embedded in a PEGylated fibrin gel followed by adipose stem cell embedded in PEGylated fibrin gel
