# Supplementary figures and images for: Delivery of silver sulfadiazine and adipose derived stem cells using fibrin hydrogel improves infected burn wound regeneration
Source: PLoS One. 2019 Jun 13;14(6):e0217965. doi: 10.1371/journal.pone.0217965 (PMC6563979; doi:10.1371/journal.pone.0217965)

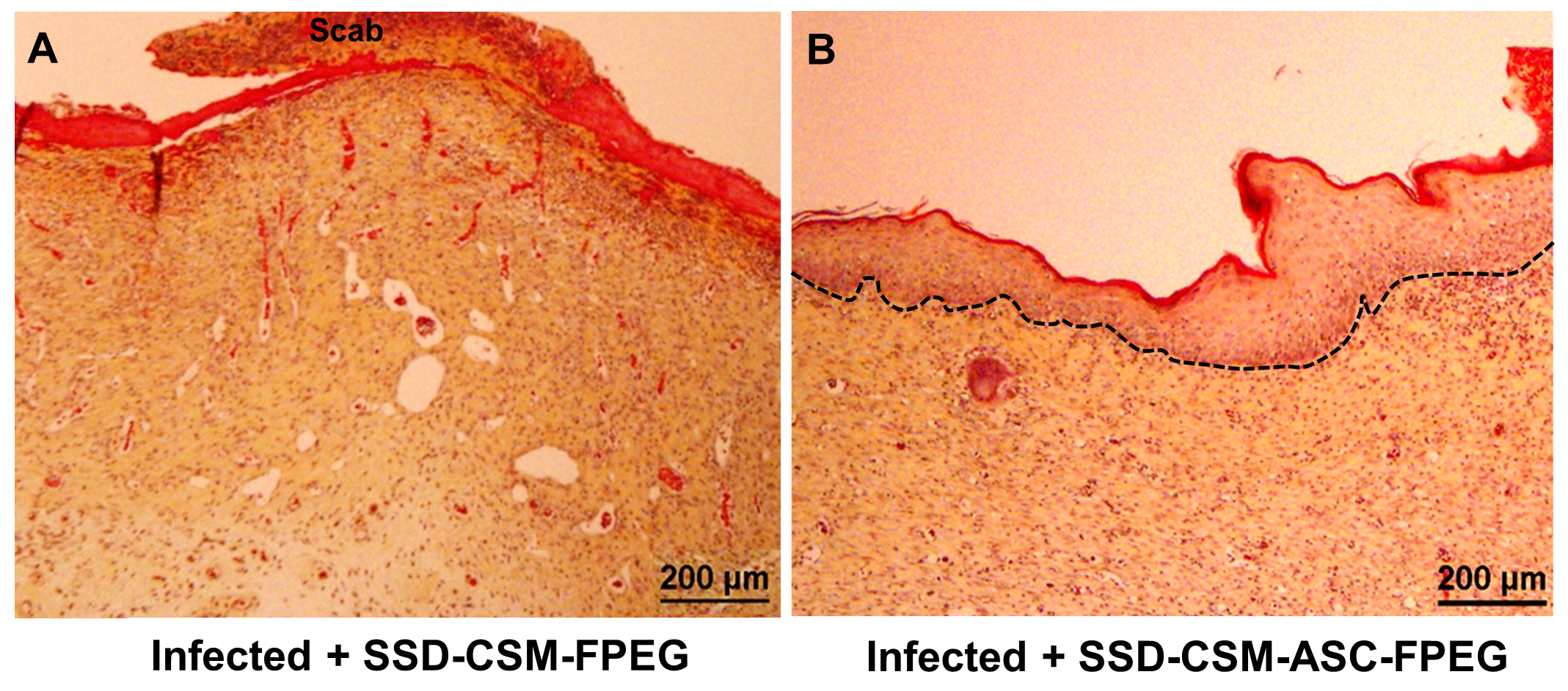

Supplement: S1 Fig — Movatt’s pentachrome stained tissue sections of wounds treated with SSDM-CSM-FPEG without (A) or with ASCs (B) on day 21. (TIF) [file pone.0217965.s001.tif]

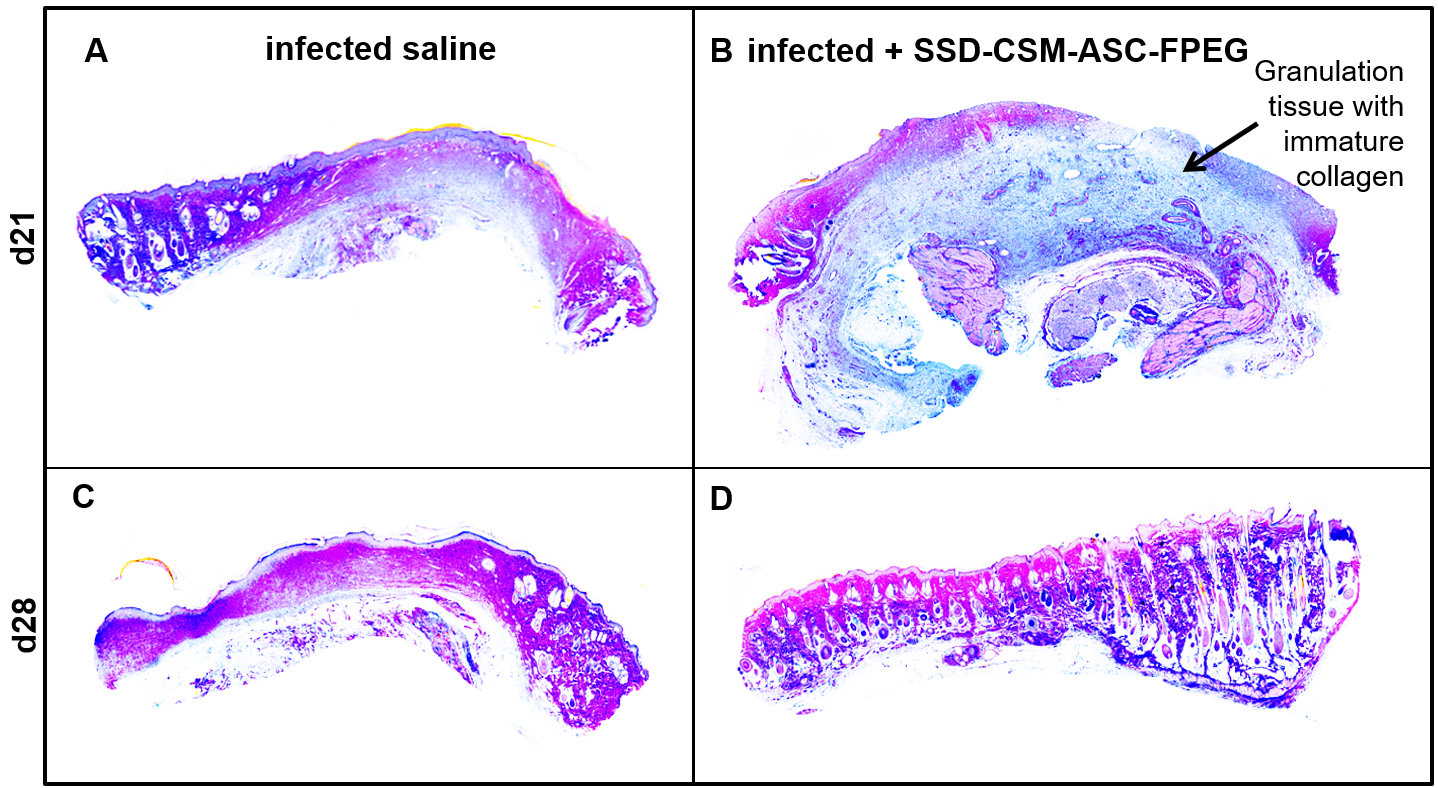

Supplement: S2 Fig — Herovici staining demonstrates thick granulation tissue with immature collagen in day 21 in SSD-CSM-ASC-FPEG treated wound samples (B) (known to support neovascularization) as compared to saline treated samples (A). On day 28, more mature collagen was observed in the sample treated with SSD-CSM-ASC-FPEG (C-D). (TIF) [file pone.0217965.s002.tif]
